# Treatment of cardiac fibrosis: from neuro-hormonal inhibitors to CAR-T cell therapy

**DOI:** 10.1007/s10741-022-10279-x

**Published:** 2022-10-11

**Authors:** Paolo Morfino, Alberto Aimo, Vincenzo Castiglione, Carolina Gálvez-Montón, Michele Emdin, Antoni Bayes-Genis

**Affiliations:** 1 Interdisciplinary Center for Health Sciences, Scuola Superiore Sant’Anna, Pisa, Italy; 2 Cardiology Division, Fondazione Toscana Gabriele Monasterio, Pisa, Italy; 3 Institut del Cor, Hospital Universitari Germans Trias I Pujol, Badalona, Spain; 4 CIBERCV, Carlos III Institute of Health, Madrid, Spain; 5 Department of Medicine, Universitat Autònoma de Barcelona, Barcelona, Spain

**Keywords:** Fibrosis, Myocardium, Anti-fibrotic therapies, Heart failure, CAR-T cells

## Abstract

Cardiac fibrosis is characterized by the deposition of extracellular matrix proteins in the spaces between cardiomyocytes following both acute and chronic tissue damage events, resulting in the remodeling and stiffening of heart tissue. Fibrosis plays an important role in the pathogenesis of many cardiovascular disorders, including heart failure and myocardial infarction. Several studies have identified fibroblasts, which are induced to differentiate into myofibroblasts in response to various types of damage, as the most important cell types involved in the fibrotic process. Some drugs, such as inhibitors of the renin–angiotensin–aldosterone system, have been shown to be effective in reducing cardiac fibrosis. There are currently no drugs with primarily anti-fibrotic action approved for clinical use, as well as the evidence of a clinical efficacy of these drugs is extremely limited, despite the numerous encouraging results from experimental studies. A new approach is represented by the use of CAR-T cells engineered in vivo using lipid nanoparticles containing mRNA coding for a receptor directed against the FAP protein, expressed by cardiac myofibroblasts. This strategy has proved to be safe and effective in reducing myocardial fibrosis and improving cardiac function in mouse models of cardiac fibrosis. Clinical studies are required to test this novel approach in humans.

Fibrosis results from excessive deposition of extracellular matrix (ECM) proteins, especially type I and III collagen, between tissue parenchymal cells. There are conditions characterized by an activation of the pro-fibrotic pathways secondary to other disease mechanisms, such as neuroendocrine activation in heart failure (HF), and conditions in which the first cause of disease is the deposition of fibrous tissue, such as idiopathic pulmonary fibrosis (IPF) and systemic sclerosis [1, 2]. In both cases, fibrosis can lead to organ dysfunction.

Tissue repair mechanisms that lead to the development of fibrosis are activated by acute or chronic tissue damage. The inflammatory response can be intense depending on the type of damage; it favors the release of mediators and the migration of neutrophils, eosinophils, and macrophages to the injured site. Tissue fibroblasts and other mesenchymal cells are activated, differentiating into myofibroblasts, which are cells with contractile action and secreting activity of ECM elements, when stimulated by pro-fibrotic cytokines such as fibroblast growth factor (FGF), platelet-derived growth factor (PDGF), and transformant-β growth factor (TGF-β) [3]. Myofibroblasts may also acquire resistance to cell death stimuli [4]. At the same time, the expression of matrix metalloproteinases (MMPs) is reduced. MMPs are mainly responsible for the replacement and degradation of ECM proteins [5].

Myocardial fibrosis is traditionally distinguished into “reparative” or “reactive” (Fig. 1). Reparative fibrosis is the replacement of necrotic cardiomyocytes following events of acute and extensive tissue damage, such as myocardial infarction (MI) or myocarditis. Other forms of myocardial damage result in reactive fibrosis, with peculiar ECM structure and composition depending on the pathogenic cause of damage (Fig. 2). For example, pressure overload is characterized by perivascular fibrosis and interstitial fibrosis due to the activation of pro-fibrotic pathways (angiotensin II and endothelin 1) [6, 7]. The two forms of fibrosis often develop together within the same disease: reparative fibrosis following MI represents the immediate tissue response to damage; then, hormonal and paracrine activation trigger the reactive fibrosis in remote regions at MI, leading to post-infarction ventricular remodeling [8]. Myocardial fibrosis also slows the conduction of the cardiac action potential and predisposes to conduction disorders such as atrioventricular block [9]. Fibrotic regions also promote the generation of re-entry circuits and can trigger focal arrhythmias. In addition, myocardial fibrosis determines a chaotic and non-linear propagation of the action potential. Fibrotic infiltration in the atrium promotes the onset of atrial fibrillation (AF), as well as impairs the compliance and thus the mechanical properties of the atrium [10, 11]. In addition, atrial fibrosis may contribute to the development of atrial amyloidosis since the accumulation of amyloid substance is accompanied by the deposition of fibrous tissue, as observed in the ventricles [12, 13].

**Fig. 1:**
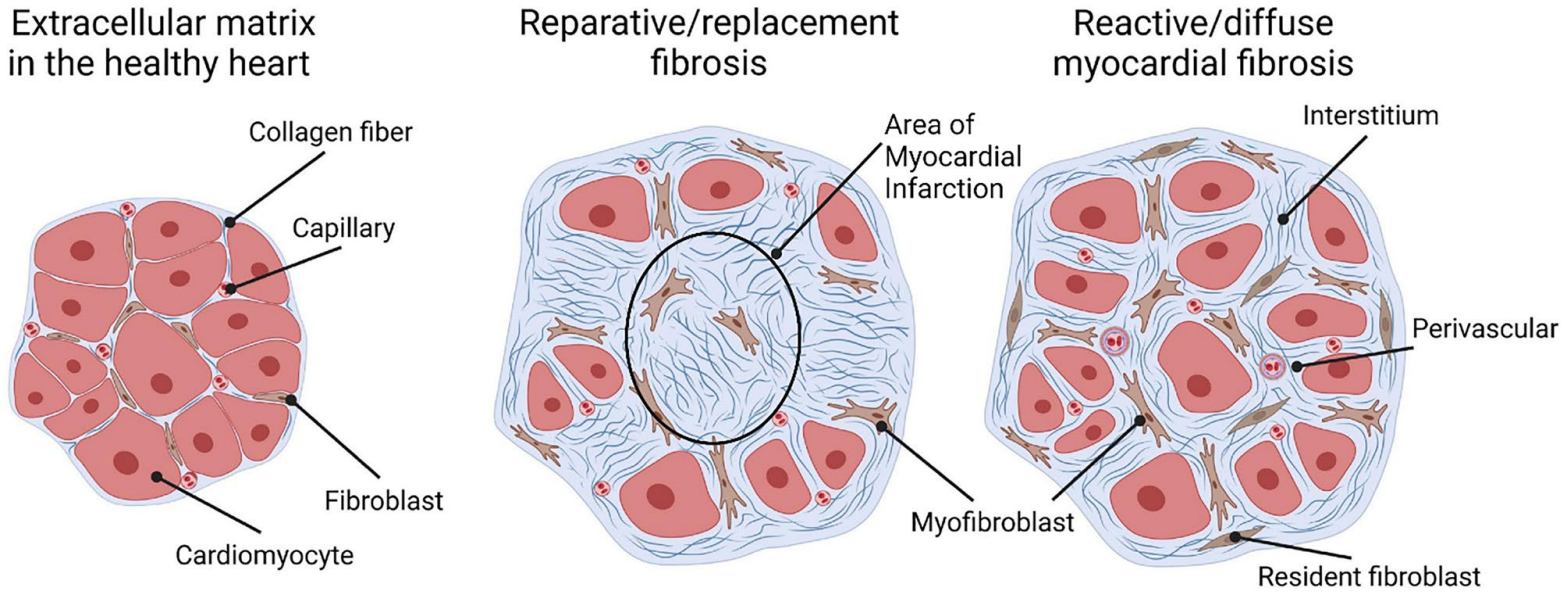
Types of cardiac fibrosis. The extracellular matrix in the healthy heart (left) is a three-dimensional network of collagen fibrils that incorporates cardiomyocytes, capillaries, and fibroblasts. The “reparative” fibrosis (center) is visible as a collagen-based scar that replaces necrotic cardiomyocytes after acute and extensive damage. “Reactive” fibrosis (right) accompanies heart failure and pressure overload, and manifests as diffuse collagen deposition in interstitial and perivascular areas. Modified with permission from Schimmel et al. [115]

**Fig. 2:**
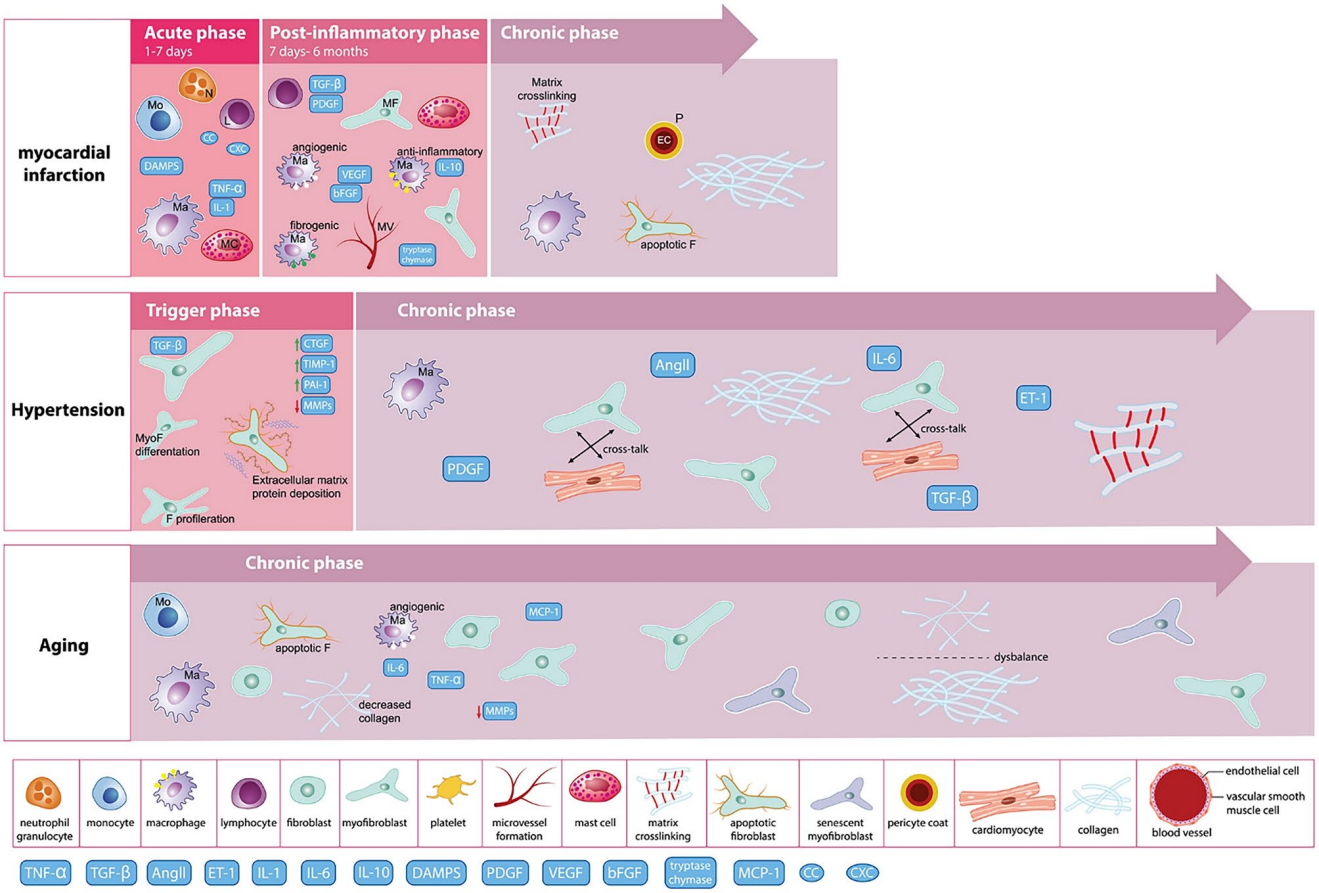
Schematic representation of heterogeneity in fibrotic progression. (Illustration: Maartje Kunen, Medical Visuals.) AngII, angiotensin II; CTGF, connective tissue growth factor; DAMPS, danger-associated molecular patterns; ET-1, endothelin-1; IL, interleukin; L, lymphocyte; Ma, macrophage; MC, mast cell; MCP-1, monocyte chemoattractant protein-1; MF/MyoF, myofibroblast; MMP, matrix metalloproteinase; MV, microvessel; N, neutrophil; PAI, plasminogen activator inhibitor; PDGF, platelet-derived growth factor; TGF, transforming growth factor; TIMP, tissue inhibitor of metalloproteinase; TNF, tumor necrosis factor. Reprinted with permission by de Boer et al. [14]

Histological analysis of endomyocardial biopsy samples with trichromatic and Sirius Red staining allows a direct evaluation of cardiac fibrosis [14, 15]. A non-invasive alternative for detecting myocardial fibrosis in vivo is cardiac magnetic resonance (CMR) imaging. Fibrotic regions are identified as late gadolinium enhancement areas. RMC also allows for the quantification of extracellular volume (ECV) [14, 16]. Regarding in vivo investigation techniques, the assay of circulating biomarkers of fibrosis is largely adopted. Among biomarkers of fibrosis, we can find collagen fragments and precursors, representing synthesis biomarkers; MMPs and MMP tissue inhibitors (TIMPs), representing degradation biomarkers; and galectin-3 and soluble tumor-2 suppressor [17]. However, recent studies show that the increase in plasma collagen biomarkers does not really reflect the extent of myocardial fibrosis, suggesting that new studies are needed for the validation of fibrosis biomarkers [18].

The amount of cardiac fibrosis has an important clinical and prognostic value. For example, there is a relationship between fibrotic extension in the left atrium at CMR and the efficacy of transcatheter ablation in patients with AF, with a greater recurrence of AF in subjects showing a larger pre-ablation fibrotic area [19]. In subjects with hypertrophic cardiomyopathy, the presence of fibrosis at CMR led to a 3.4-fold higher risk of mortality or hospitalization due to cardiovascular causes or implantation of cardioverter-defibrillator, with a 15% increase in risk as a result of a 5% increase in fibrosis; whereas in subjects with acute coronary syndrome the presence of cardiac fibrosis at CMR is strongly associated with sudden cardiac death [20, 21]. For these reasons, myocardial fibrosis can be considered an important therapeutic target.

Some drugs, such as renin–angiotensin–aldosterone system (RAAS) inhibitors, have been shown to be effective in reducing ECM deposition in the myocardium. However, no primarily anti-fibrotic medications have been approved for the treatment of cardiovascular disease [22]. Studies on animal models have revealed a wide variety of effective molecules in the treatment of fibrosis, but there is still no evidence of clinical benefit (Table 1). Recently, the first results from the phase II clinical trial PIROUETTE (The Efficacy and Safety of Pirfenidone in Patients with Heart Failure and Preserved Left Ventricular Ejection Fraction) show that pirfenidone may be effective and safe in patients with HF and preserved ejection fraction (FE) [23]. Anti-fibrotic T-cell therapy with chimeric antigen receptor (CAR) is widely used in oncology. Recent studies on preclinical models also encourage their potential use in cardiovascular diseases [24].

**Table 1:** Main evidence of anti-fibrotic drugs from clinical studies

**Study**	**Therapy**	**Follow-up**	**Included patients (*n*)**	**Main evidence**	
**RAAS inhibitors**					
Brilla et al. [27]	Lisinopril	6 months	35	Lisinopril reduces CVF in hypertensive patients compared with hydrochlorothiazide diuretic	
López et al. [28]	Losartan	12 months	37	Losartan reduces CVF and PICP in hypertensive patients compared with amlodipine	
Díez et al. [29]	Losartan	12 months	19	Losartan reduces CVF and LV stiffness in hypertensive patients with severe fibrosis	
Shimada et al. [30]	Losartan	12 months	20	Losartan reduces fibrotic progression in patients with nonobstructive hypertrophic CMP	
Kosmala et al. [32]	Spironolactone	6 months	80	ACEi and spironolactone reduce PICP and PIIINP in patients with metabolic syndrome compared with ACEi alone	
Kosmala et al. [33]	Spironolactone	6 months	113	Spironolactone improves myocardial deformation and reduces PICP and PIIINP in obese patients with mild diastolic dysfunction	
Mak et al. [34]	Eplerenone	12 months	44	Eplerenone reduces PIIINP and slightly improves diastolic function in patients with diastolic HF	
Deswal et al. [35]	Eplerenone	6 months	44	Eplerenone reduces PINP and PICP in patients with HFpEF	
Zannad et al. (post-hoc analysis RALES trial) [36]	Spironolactone	6 months	261	Spironolactone reduces PICP, PINP, and PIIINP in patients with HFrEF	
Iraqi et al. (post hoc analysis EPHESUS trial) [38]	Eplerenone	6 months	476	Eplerenone reduces PINP and PIIINP in patients with post-MI diastolic HF	
Ravassa et al. (post hoc analysis ALDO-DHF trial) [39]	Spironolactone	12 months	381	Spironolactone reduces PICP and improves diastolic function in patients with HFpEF. Patients with CITP/MMP-1 < 2.5 show less benefit from treatment	
Cunningham et al. (post hoc analysis PARAMOUNT trial) [45]	Sacubitril/valsartan	16 weeks	1113	Sacubitril/valsartan reduces TIMP-1, sST2, and PIIINP and increases CITP in HFpEF patients compared to valsartan alone	
**Inflammation modulators**					
RENEWAL trial [47]	Etanercept	6 months	2356	Etanercept does not impact on mortality and hospitalizations in patients with HF	
ATTACH trial [48]	Infliximab	Discontinued	150	Infliximab increases mortality in patients with HF	
COLCOT trial [53]	Colchicine	22.6 months	4745	Colchicine reduces the risk of ischemic cardiovascular events in patients with MI to 30 days	
COVERT-MI [55]	Colchicine	3 months	192	Colchicine improves the size of the infarcted area after MI and increases the risk of thrombus in LV	
Abulhul et al. [62]	Atorvastatin	6 months	56	Atorvastatin reduces PIIINP levels in patients with systolic HF and normal cholesterol	
Chang et al. [63]	Atorvastatin	12 weeks	15	Atorvastatin reduces PIIINP and TIMP-1 in hypertensive patients with atherosclerosis	
Ashton et al. (post-hoc analysis UNIVERSE trial) [64]	Rosuvastatin	6 months	32	Rosuvastatin increases PINP and PIIINP in patients with chronic HF	
GISSI-HF [66]	Rosuvastatin	3.9 years	4574	Rosuvastatin does not improve the prognosis of patients with HF	
CORONA [65]	Rosuvastatin	32.8 months	5011	Rosuvastatin does not improve the prognosis of patients with systolic HF	
**TGF-β signaling inhibitors**					
AlAnsari et al. [80]	Pirfenidone	Retrospective study	27	Pirfenidone does not modify echocardiographic parameters in patients with IPF	
AlAnsari et al. [81]	Pirfenidone	Retrospective study	24	Pirfenidone reduces the telesistolic and telediastolic LV volumes in patients with HFpEF and IPF	
PIROUETTE trial [23]	Pirfenidone	52 weeks	94	Pirfenidone slightly reduces ECV, although it does not change diastolic function parameters, in patients with HFpEF	
**MMP inhibitors**					
PREMIER trial [84]	PG-116800	90 days	253	PG-116800 does not prevent LV remodeling nor improve the mortality rate and re-infarction in patients with MI	
**β3-AR modulators**					
BEAT-HF trial [96]	Mirabegron	6 months	70	Mirabegron does not improve cardiac function in terms of LVEF in patients with HFrEF	

This literature review summarizes the possible options to target myocardial fibrosis, including the new perspective of CAR-T cell therapy.

## Drugs with no primarily anti-fibrotic role

### RAAS inhibitors

Angiotensin II (Ang II) binding Ang II type 1 receptors (AT1R) promotes collagen synthesis [25]. In chronic heart disease, there is generally a significant activation of RAAS, which is directly associated with the development of cardiac fibrosis [26]. Various studies have shown that both angiotensin-converting enzyme (ACE) inhibitors and angiotensin II receptor blockers (ARBs) significantly reduce myocardial fibrosis regardless of their hypotensive effect. In a sample of hypertensive patients treated with lisinopril (*n* = 18), endomyocardial biopsy at 6 months revealed a significant reduction in collagen volume fraction (CVF) compared to patients treated with hydrochlorothiazide diuretic (*n* = 17) [27]. In a study with hypertensive patients treated with losartan (*n* = 21) and amlodipine (*n* = 16) with 1-year follow-up, the first group showed reduction of the collagen fraction and the carboxy-terminal pro-peptide of type 1 pro-collagen (PICP) [28]. Another study showed the effectiveness of losartan in reducing CVF and increasing left ventricular (LV) compliance to 1 year, but only in patients with severe fibrosis (CVF > 6%, *n* = 7) [29]. A further study showed attenuation in the progression of cardiac fibrosis in patients with nonobstructive hypertrophic cardiomyopathy treated with losartan [30].

Aldosterone, whose production is stimulated by Ang II, also exerts a pro-fibrotic effect in the myocardium by interacting with mineralocorticoid receptors [31]. Aldosterone receptor antagonists (spironolactone, canrenone, and eplerenone) showed significant anti-fibrotic effects. In 80 patients with metabolic syndrome treated with ACE inhibitors (ACEi), treatment with spironolactone for 6 months improved diastolic function and decreased levels of PICP and amino-terminal pro-peptide of type III pro-collagen (PIIINP) [32]. Similar results were obtained in another study which included 113 patients with obesity and mild diastolic dysfunction [33]. In 44 patients with HF with preserved EF (HFpEF), the administration of eplerenone for 12 months reduced PIIINP levels and resulted in a modest improvement in diastolic function [34]. Similar results emerged in a study showing that eplerenone reduced the PINP and PICP levels in 44 patients with HFpEF [35]. In the RALES study (Randomized Aldactone Evaluation Study), conducted in patients with HF with reduced EF (HFrEF), spironolactone was associated with reduced mortality and hospitalization, as well as with reduction in blood levels of fibrosis biomarkers and collagen synthesis [36, 37]. In a sub-analysis of the study EPHESUS (the Eplerenone Post-Acute Myocardial Infarction Heart Failure Efficacy and Survival Study), which evaluated the effect of eplerenone in patients with HF after MI (*n* = 476), treatment with eplerenone showed a significant reduction in plasma levels of PINP and PIIINP (*p* < 0.007 at 6 months for both), although these were not associated with prognostic events (mortality and hospitalization) [38]. Treatment with eplerenone (25 mg/day up to 50 mg/day, treated *n* = 3313, placebo *n* = 3319) was also associated with a reduction in the risk of mortality and hospitalization for all causes (relative risk (RR): 0.92; 95% CI: [0.86; 0.98]; *p* = 0.02) after 16 months in treated patients with MI complicated by subsequent LV and cardiac dysfunction compared to controls [38]. A sub-study of the ALDO-DHF trial (The Aldosterone Receptor Blockade in Diastolic Heart Failure), which included 381 patients with HFpEF, identified that treatment with spironolactone reduces PICP levels and improves diastolic function after 12 months of treatment [39].

Several studies also highlight that the type B natriuretic peptide (BNP) plays an anti-fibrotic role through the interaction with its NPR-A and NPR-B receptors and subsequent activation of cGMP-dependent kinase (PKG) [40]. Although the role of the cGMP pathway in fibrosis is not fully understood, several preclinical studies have shown that an increase in PKG levels plays an anti-fibrotic effect through negative interference with the TGF-β pathway, which plays a crucial role in the activation of fibroblasts [41–44]. The administration of sacubitril/valsartan, which represents an association of ARB and an inhibitor of neprilysin, which is the BNP degradation enzyme, has been shown to be effective in reducing fibrosis. PARAMOUNT (Prospective Comparison of ARNI With ARB on Management of Heart Failure with Preserved Ejection Fraction) trial showed that treatment with sacubitril/valsartan leads to the reduction of plasma biomarkers of cardiac fibrosis in patients with HFpEF [45]. Sixteen weeks after administration, the treatment group showed a reduction in plasma levels of TIMP-1 (8%; 95% CI: [6; 10%]; *p* < 0.001), soluble suppression of tumorigenicity 2 (sST2) (4%; 95% CI: [1; 7%]; *p* = 0.002) and PIIINP (3%; 95% CI: [0; 6%]; *p* = 0.04), and an increase in carboxy-terminal telopeptide of type I collagen (CITP) (4%; 95% CI: [1 to 8%]; *p* = 0.02), compared to patients treated with valsartan alone. This additional benefit seems to be caused by the increase in BNP levels [45].

### Inflammation modulators

Tissue damage triggers a phlogistic process that triggers the deposition of fibrotic tissue. Tumor necrosis factor α (TNF-α) plays an important role in stimulating cardiac fibrosis [46]. However, the RENEWAL (Randomized etanercept Worldwide evaluation) study, which evaluated the effect of the TNF-α antagonist etanercept in patients with HF, showed no benefit in terms of mortality and hospitalization [47]. The ATTACH (anti-TNF Therapy Against Congestive Heart failure) study was prematurely discontinued due to increased mortality in patients with HF on infliximab, a TNF-α antagonist [48]. The later discovery that TNF-1 and TNF-2 receptors have opposite effects on cardiac remodeling may partly explain the disappointing results of TNF-α inhibition [49].

Colchicine has an important anti-inflammatory action because of its effectiveness in inhibiting inflammasome network, various pro-inflammatory cytokines and chemokines [50]. In mouse models of MI, colchicine has been shown to be effective in reducing the extent of the infarcted area. The reduction in the extent of fibrosis has been confirmed in a study on rabbit with HF [51, 52]. The COLCOT (COLCHICINE Cardiovascular Outcomes Trial) study, which randomized 4745 patients with MI to colchicine or placebo, revealed a lower risk of ischemic cardiovascular events at 30 days from MI in the treated group [53, 54]. The effect on myocardial fibrosis has not been specifically assessed. The recent COVERT-MI study (colchicine for Left ventricular Remodeling Treatment in Acute Myocardial Infarction) revealed that patients treated with colchicine after MI (*n* = 101) showed no difference in the size of the infarcted area at CMR compared to the controls (*n* = 91) [55].

Peroxisome-α proliferator-activated receptor (PPAR-α) agonists reduced myocardial fibrosis and improved cardiac function in animal models [56]. On the other hand, excessive expression of PPAR-α is associated with lipotoxicity, ventricular dysfunction, and cardiac hypertrophy [57, 58].

Besides reducing cholesterol levels, statins have a powerful anti-inflammatory and cardioprotective action by inhibiting the proteins Ras, Rho, and NF-kB, and activating the PI3K/Akt/Enos pathway [59, 60]. Rosuvastatin has been shown to be effective in attenuating cardiac fibrosis in mouse models of hypertensive heart disease [61]. In a small clinical study, therapy with 40 mg/day of atorvastatin for 6 months reduced PIIINP levels (4.65 ± 1.86 to 4.09 ± 1.25 ng/ml, *p* < 0.05) in patients with systolic HF and normal cholesterol (*n* = 56) [62]. A study on hypertensive patients with atherosclerosis (*n* = 15) treated for the first time with 40 mg/day of atorvastatin for 12 weeks confirmed the effectiveness of the drug in reducing plasma levels of PIIINP (9.5 ± 2.7 to 6.4 ± 1.4 ng/ml, *p* = 0.012) and TIMP-1 (299 ± 65 to 250 ± 45 ng/ml, *p* = 0.024) [63]. Conflicting results were obtained in a sub-study of the UNIVERSE trial (The rosuvastatin Impact on VEentricular Remodeling cytokineS and neurohormonEs), in which an increase in plasma markers of collagen PINP (*p* = 0.03) and PIIINP (*p* = 0.001) was observed in patients with chronic HF (*n* = 32) treated for 6 months with rosuvastatin titrated up to 40 mg/day [64]. In conclusion, the role of statins in the treatment of chronic HF is still controversial. Although several retrospective studies have revealed a better prognosis in patients with HF treated with statins, two randomized clinical trials, GISSI-HF (Italian Group for the Study of Survival in Heart Failure Failure) and CORONA (Controlled Rosuvastatin Multinational Trial in Heart Failure), reported no prognostic benefit from rosuvastatin treatment in this class of patients [65, 66].

Overall, there is no clear evidence that anti-inflammatory drugs are effective in reducing cardiac fibrosis.

### Anti-TGF-β antibodies

**Fig. 3:**
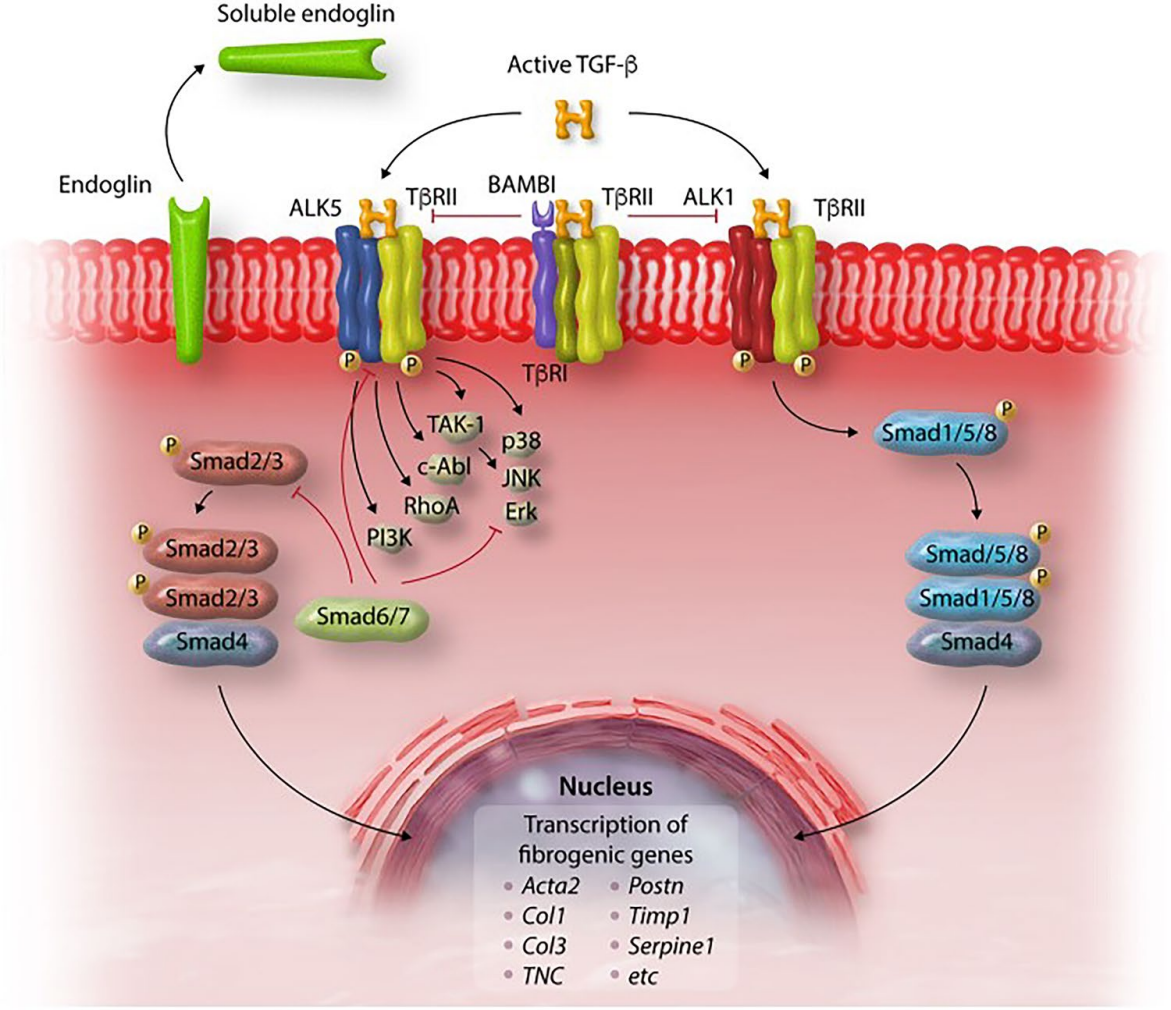
Regulation of TGF-β signaling in cardiac fibrosis. Active TGF-β binds to type II and type I receptors, activating downstream Smad-dependent signaling cascades and Smad-independent pathways. TGF-β binding to the ALK5 type 1 receptor and downstream activation of Smad3 signaling induces a matrix-preserving program in cardiac fibroblasts and plays an important role in their activation following cardiac injury. In contrast, the role of ALK1/Smad1/5 signaling in regulation of fibroblast phenotype is poorly understood. Activation of Smad-independent pathways, including RhoA and MAPK signaling, mediates some of the effects of TGF-b in cardiac fibroblasts. Endogenous pathways for negative regulation of TGF-b cascades may protect from excessive or unrestrained fibrotic responses. The inhibitory Smads (Smad6/7), pseudoreceptors such as BAMBI, and soluble endoglin may serve as endogenous inhibitors of TGF-b signaling, limiting pro-fibrotic responses. Reprinted with permission from Frangogiannis [6]

Transforming-β growth factor (TGF-β) has a central role in the development of cardiac fibrosis. TGF-β achieves its pro-fibrotic effect by the ALK/Smad2/3/Smad4, TAK/p-38/JNK, and NOX4/ROS signaling pathways (Fig. 3) [42, 67]. In mouse models of MI and hypertensive heart disease, anti-TGF-β and ALK5 antibodies led to reduction of myocardial fibrosis but not cardiomyocyte hypertrophy. However, anti-TGF-β antibody therapy has also been associated with serious adverse effects, including LV dilation and increased mortality [68, 69]. The blockade of the TGF-β signaling pathway through antibodies therefore seems dangerous, while less intense inhibition may be more effective.

### Pirfenidone

Pirfenidone is an oral anti-fibrotic drug approved for the treatment of IPF [70, 71]. Since pulmonary and cardiac fibrosis share many pathophysiological mechanisms, there is an increasing interest in the application of pirfenidone in cardiovascular diseases [72]. The mechanism of action of pirfenidone remains to be elucidated, but it seems to reduce the expression of pro-fibrotic factors such as TGF-β and pro-inflammatory cytokines such as TNF-α, interleukin (IL)-4, and IL-13 [73]. Pirfenidone also promotes MMPs expression with subsequent reduction of ECM protein accumulation [74]. Pirfenidone could also contribute to the modulation of activation and proliferation of T and B cells, thus regulating the secretion of numerous pro-inflammatory and pro-fibrotic molecules, such as TNF-α and TGF-β [75–77].

In mouse models of hypertension, the administration of pirfenidone has been associated with reduced LV hypertrophy and increased survival compared to controls [78]. Another study documented that pirfenidone reduces ventricular remodeling and hinders interstitial fibrosis induced by Ang II infusion [79].

Only two retrospective studies evaluated the efficacy of pirfenidone on cardiac parameters in patients with IPF. In the first, no association between administration of pirfenidone and change in echocardiographic parameters was found [80]. In the second, pirfenidone was associated with a reduction in end-systolic and end-diastolic LV volumes, but without significant changes in ventricular function [81]. The PIROUETTE phase II study included 94 patients with HFpEF and extended fibrosis, defined as 27% ECV [23]. After a 52-week follow-up, an absolute reduction of 0.7% in the ECV in pirfenidone-treated group was found compared to an increase of 0.5% in placebo-treated controls (*p* = 0.009). This very limited effect on the ECV was not associated with significant changes in diastolic function parameters [23].

### MMP inhibitors

Although MMP inhibitors (MMPi) have been shown to attenuate cardiac fibrosis and remodeling in experimental models, these results have not achieved the expected clinical results [82, 83]. In the PREMIER (Prevention of Myocardial Infarction Early Remodeling) study, in which the effect of oral administration of PG-116800 in 253 patients with recent MI was evaluated, the drug has not shown any beneficial effect in preventing LV remodeling or improving the mortality rate or re-infarction at 90 days after MI [84].

### Adrenergic receptor system modulators

Pharmacological blockade of β1 adrenergic receptors (β1-ARs) is a fundamental therapy for the treatment of HF and for the prevention of structural remodeling, preventing and attenuating progressive dilation of LV and cardiac hypertrophy [85–87]. In contrast, the signaling pathway of β2-AR plays anti-fibrotic effects. However, while the acute effect of β2-AR activation inhibits collagen synthesis, myofibroblasts extracted and isolated from patients with HF appear to be resistant to β2-AR agonists, possibly due to the increased activity of GPCR-2 kinase (GRK2) [88]. In fact, numerous studies on mouse models have highlighted the pathological role of GRK2 in HF. GRK2 became an attractive pharmacological target after the discovery that its inhibition is associated with significant protection against myocardial fibrosis in HF animal models [89]. In a pig model of post-MI HF, adeno-associated virus gene therapy allowed the expression of a GRK2 inhibitor peptide (βARKct), demonstrating long-term improvement of heart function [90].

Unlike β1- and β2-AR, β3-AR is thought to be resistant to desensitization because it lacks phosphorylation sites for GRK kinases [91]. Cardiac expression of β3-AR is physiologically low, but it increases in chronic disease conditions [92]. Evidence of the cardioprotective role of β3-ARs emerged from the demonstration that mice without β3-ARs experienced significant cardiac remodeling in response to transverse aortic constriction (TAC) [93]. β3-AR agonists (BRLs) have proven effective in counteracting cardiac fibrosis in several preclinical models of cardiovascular disease. In murine models of HFpEF induced by infusion of Ang II, the administration of BRLs has shown beneficial effects associated with attenuation of cardiac fibrosis, including improvement of myocardial stiffness and reduction of pulmonary congestion [94]. In mice knockout for neuronal nitric oxide synthase (nNOS) undergoing TAC, the administration of BRLs for 3 weeks revealed attenuation of ventricular dilation, systolic dysfunction, and partial reduction of cardiac hypertrophy [95]. However, the first randomized controlled trial that evaluated the effectiveness of the β3-AR mirabegron agonist in the HFrEF, namely, the BEAT-HF trial (Baroreflex Activation Therapy for Heart Failure), showed no improvement in cardiac function in terms of EF variation over a period of 6 months [96].

## Anti-fibrotic therapy with CAR-T cells

### Ex vivo engineered CAR-T cell therapy

CARs are engineered receptors that function to redirect lymphocytes, most commonly T cells, to recognize and eliminate cells expressing a specific target antigen. This interaction occurs in a specific CAR domain called “antigen binding domain” and allows endogenous activation of T cells, with subsequent elimination of target cells [97, 98]. CAR-T cell therapy represents a revolutionary therapeutic approach with significant and lasting clinical benefits already demonstrated in hematological diseases, such as large B-cell lymphoma or acute lymphoblastic leukemia (ALL) [99]. CAR-T cell therapy was approved by the Food and Drug Administration (FDA) in 2017 for the treatment of ALL [100].

Aghajanian et al. firstly investigated the effectiveness of ex vivo engineered T cells for the selective elimination of myofibroblasts expressing the fibroblast activation protein (FAP) on the membrane surface, in a mouse model of fibrosis induced by infusion of Ang II and phenylephrine [101]. The authors identified FAP as the best marker of cardiac myofibroblasts since peptide expression is almost exclusive to this cell population. The use of CAR-T cells directed against FAP resulted in the reduction of fibrosis in all seven treated mice, and an almost total elimination in 5/7, as well as the maintenance of normal systolic and diastolic function [101]. A confirmation of the selectivity of CAR-T cells is provided by the persistence of perivascular fibrosis after treatment since perivascular fibroblasts do not expose FAP on the membrane surface and therefore do not represent a target for CAR-T FAP cells. In addition, the administration of engineered T cells in mice with fibrosis was shown to be safe and poorly cardiotoxic, with a very mild inflammatory response [101].

**Fig. 4:**
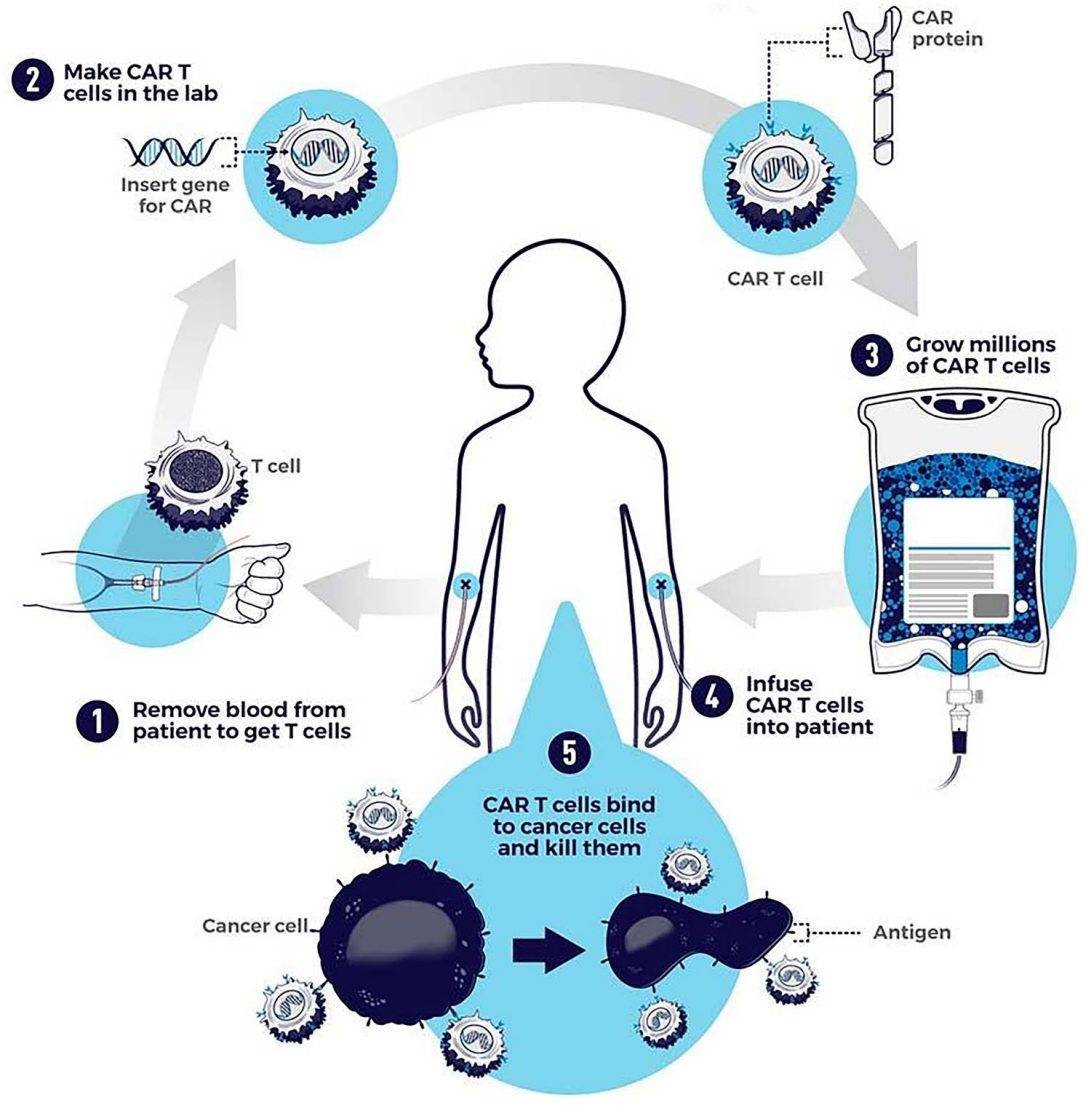
Ex vivo production of CAR-T cells. Autologous T cells are extracted from the patient, then they are engineered in the laboratory to obtain CAR expression and the differentiation of T lymphocytes into CAR-T cells, which will then be amplified and re-infused into the patient with prior lymphodepletion. Reprinted with permission from https://www.cancer.gov/about-cancer/treatment/research/car-t-cells

CAR-T cells are usually produced ex vivo by transduction using a retrovirus or lentivirus containing a DNA or RNA coding for a genetically modified CAR protein, in autologous T cells taken from the patient. Once modified, the T cells are expanded and infused into the patient with prior depletion of naïve T cells (Fig. 4) [102]. This method is effective but long and expensive. A further limitation of ex vivo engineered CAR-T cells is their persistent activation after infusion [103].

The most common reported adverse effect is the syndrome of release of pro-inflammatory cytokines by activated CAR-T cells and other immune cells, with an incidence of mild complications of 70–90% and severe complications, such as cardiogenic shock and multiorgan failure, equal to 20–50% [104, 105]. Potential toxicity associated with CAR-T cell therapy has stimulated the search for alternative approaches, such as the use of CAR-Natural Killer (NK) cells, and safer cell programming methods.

### Use of CAR-T cells for the treatment of myocardial fibrosis *in vivo*

Rurik et al. developed a strategy to obtain anti-fibrotic transient CAR-T cells using a system of lipid nanoparticles (LNPs) that enclose modified mRNA [24]. The introduction of mRNA into T cells to transform them into CAR-T cells had already been performed through electroporation method, but exclusively ex vivo [106]. To avoid the need for extraction and reinfusion of T cells, the authors have developed an approach to achieve in vivo differentiation of CAR-T cells by infusion of LNP containing modified mRNA. The use of LNP-mRNA systems has been very successful in the production of vaccines against COVID-19 and in other clinical contexts since LNPs can be coated with antibodies directed against a specific target cell, such as T cells, to which they release the mRNA [107]. The authors managed to produce CAR-T cells less stable and durable than that normally produced ex vivo, avoiding the problem of their indefinite permanence and activation.

**Fig. 5:**
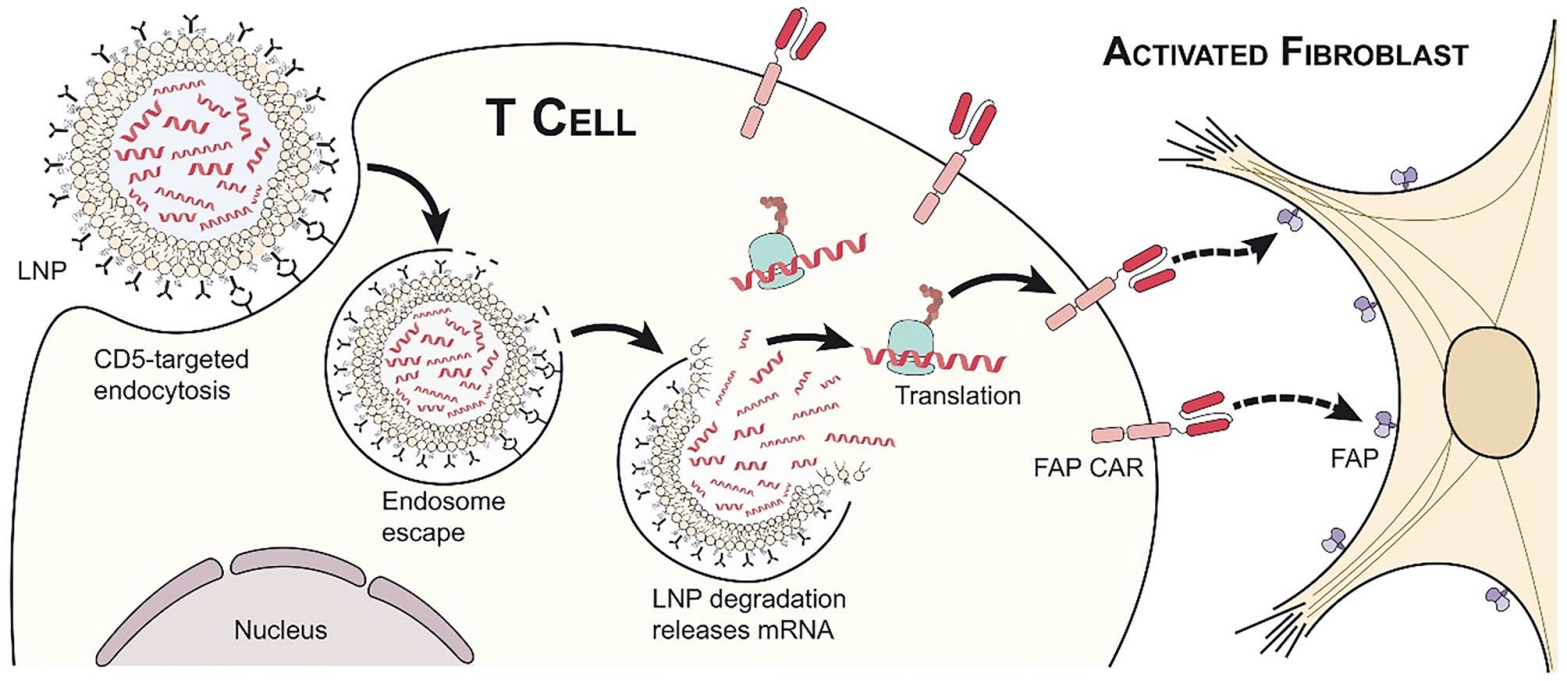
In vivo production of CAR-T cells using CD5/LNP-FAPCAR. Administration of LNP coated with anti-CD5 antibodies and containing mRNA coding for the FAPCAR membrane receptor, which selectively recognizes the FAP protein expressed by cardiac myofibroblasts, allows to obtain transients CAR-T cells in vivo that specifically eliminate the pro-fibrotic cells from the injured myocardium. CAR, chimeric antigen receptor; FAP, fibroblast activation protein; LNP, lipid nanoparticle. Reprinted with permission from Rurik et al. [24]

Ex vivo production of CAR-T cells consists in the transduction with viral vector and the integration of a fragment of nucleic acid coding for the engineered CAR protein in the genome of the target cell. On contrast, the in vivo infusion through LNP-mRNA system leads to endocytosis of the LNP, resulting in the degradation and thus the release of mRNA, which is incapable of genomic integration and has a limited half-life, into the T cells’ cytoplasm [24] (Fig. 5).

**Fig. 6:**
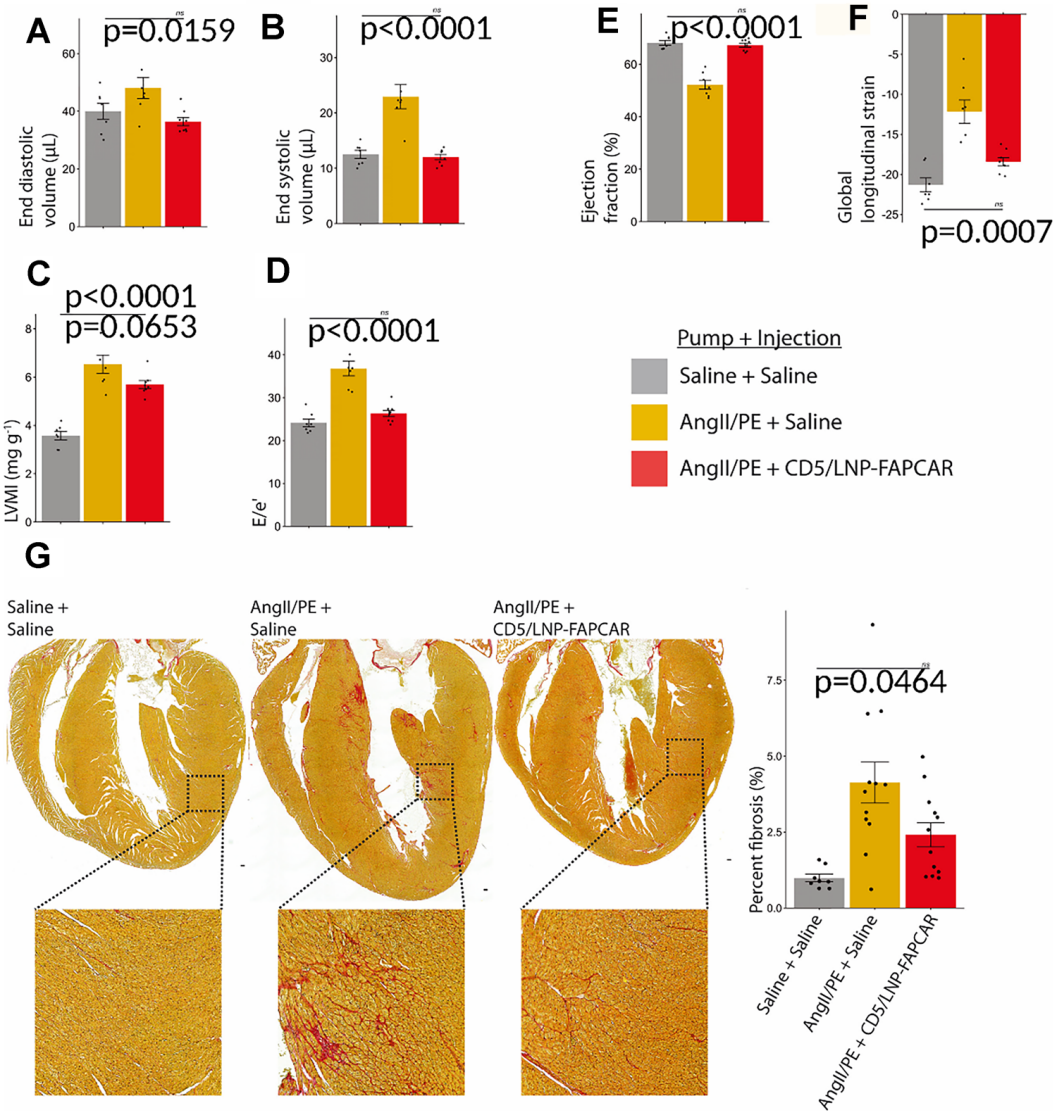
In vivo engineered CAR-T cells against FAP improve cardiac function after myocardial damage. Adult wild-type mice C57BL/6 received a continuous infusion with saline or Ang II + PE via mini-osmotic pump implanted for 28 days. After a week of heart damage due to pressure overload, mice received a single dose of 10 mg of CD5/LNP-FAPCAR. Mice were analyzed 2 weeks after treatment. Telediastolic (A) and telesystolic (B) volume measurement of LV. Left Ventricular Mass Index (LVMI) (C), diastolic function (E/e ratio) (D), EF (E), and global longitudinal strain (F) estimation. Picrosirius red staining (G) highlights collagen (pink) in coronal section of uninjured mice (*n* = 8, 3 weeks after saline infusion pump implantation + 1-week saline injection), damaged control mice (*n* = 11, Ang II + PE + saline), and damaged treated mice (*n* = 12, Ang II + PE + CD5/LNP-FAPCAR). The quantification of fibrosis is expressed as a percentage of the observed ventricle. The data are expressed as average ± standard error. The *p* values shown derive from Tukey’s post hoc test after one-way ANOVA (*p* < 0.05). Ang II, angiotensin II; EF, ejection fraction; PE, phenylephrine. Modified with permission from Rurik et al. [24]

Rurik et al. first designed a modified mRNA coding for a CAR receptor directed against the FAP protein (FAPCAR), expressed by cardiac fibroblasts activated by tissue damage, and encapsulated it within an anti-CD5 antibody-coated LNP (CD5/LNP-FAPCAR). CD5 is a membrane glycoprotein physiologically expressed by T cells [108]. The authors subsequently tested the effectiveness of the CD5/LNP-FAPCAR system in transforming a culture of T cells into CAR-T cells, and their ability to eliminate target cells with FAP expression in vitro. The positive results encouraged the translation of the method in vivo. Mice injected with Ang II and phenylephrine for 28 days then received intravenous administration of CD5/LNP-FAPCAR [109]. At 48 h after the injection, the authors detected a significant expression of FAPCAR in a portion (17.5–24.7%) of T cells (FAPCAR + -T), in the treated group [24]. Treated mice showed improved size, systolic and diastolic function, and LV mass compared to untreated mice [24] (Fig. 6). Histological analysis showed a significant reduction in the ECM fraction, with regression of interstitial fibrosis, so that 5 of the 12 samples of treated mice were indistinguishable from sham animals [24].

Overall, the use of in vivo engineered CAR-T cells via LNP-mRNA and directed against FAP results in the elimination of cardiac myofibroblasts, in the reduction of myocardial fibrosis and improvement of cardiac geometry and function, potentially exceeding the limits of ex vivo engineering of CAR-T cells.

### Feasibility of CAR-T cell therapy in humans

The infusion of CAR-T cells is emerging as a good approach to counteract cancer development and the number of potential targets suitable for CAR-T cell therapy is growing rapidly. The heart and the immune system are highly cross-linked and accumulating data suggest that the modulation of immunological cells may provide beneficial effects in many cardiovascular diseases, such as cardiac fibrosis, coronary atherosclerosis, and HF [110, 111]. The successful application of CAR-T cells to cancer treatment could be thus translated to other fields, making the CAR-T cells immunotherapy a potential approach against cardiovascular diseases.

However, there are still important limitations to CAR-T cell therapy that need to be solved. One of the most common limitations of CAR-T cell therapy in oncological disease is the development of the so-called antigen escape, which is the acquired tissue resistance to single antigen targeting. The cells of a significant portion of patients treated with CAR-T cells may partly or completely lose the target antigen expression over time, thus eluding the CAR-T cells [112]. Moreover, CAR-T cell therapy is highly associated with life-threatening toxicities and cardiovascular events such as symptomatic heart failure, acute coronary syndrome, ischemic stroke, and de novo cardiac arrhythmia [113]. The toxic effects of CAR-T cells on cardiovascular system need to be further elucidated [113, 114]. In conclusion, new strategies are emerging, and they may provide a path forward more effective and safer future applications of CAR-T cell immunotherapy in cardiovascular diseases.

## Conclusion

The pathophysiological heterogeneity of myocardial fibrosis and the complexity of tissue fibroblasts’ response to induced damage complicate the development of anti-fibrotic strategies in cardiovascular diseases. Some drugs have proven effective in reducing further deposition of ECM in the myocardium, such as RAAS inhibitors. Various purely anti-fibrotic strategies have performed promising results in preclinical models of cardiovascular disease. However, there is currently no anti-fibrotic drug that has clearly demonstrated the regression of fibrosis and the health improvement in clinical trials. These results encourage the need for a new approach, potentially directed against the myofibroblasts, namely, the cells responsible for the fibrotic response, rather than on single molecular pathways. In vivo engineered CAR-T cells therapy is a novel approach, particularly promising for the treatment of pathological conditions characterized by an intense activation of pro-fibrotic pathways (such as MI or myocarditis). The effectiveness and safety of this approach should be verified in dedicated clinical trials.

*ACEi* angiotensin-converting enzyme inhibitor, *CITP* carboxy-terminal telopeptide of type I collagen, *CMP* cardiomyopathy, *CVF* collagen volume fraction, *ECV* extracellular volume, *EF* ejection fraction, *IPF* idiopathic pulmonary fibrosis, *HFpEF* heart failure with preserved ejection fraction, *HFrEF* heart failure with reduced ejection fraction, *MI* myocardial infarction, *MMP* matrix metalloproteinase, *PICP* carboxy-terminal pro-peptide of type I pro-collagen, *PINP* amino-terminal pro-peptide of type I pro-collagen, *PIIINP* amino-terminal pro-peptide of type III pro-collagen, *HF* heart failure, *sST2* soluble suppression of tumorigenicity 2, *LV* left ventricle, *TIMP-1* tissue inhibitor of MMP-1.
